# Bayesian Mendelian randomization reveals a protective effect of later age at first sexual intercourse against erectile dysfunction: Erratum

**DOI:** 10.1097/MD.0000000000050687

**Published:** 2026-09-04

**Authors:** Jiani Wu, Xueming Hui

In the article “Bayesian Mendelian randomization reveals a protective effect of later age at first sexual intercourse against erectile dysfunction,”^[1]^ which appears in Volume 105, Issue 24 of *Medicine*, Tables 2 and 4 incorrectly state the prior for Group 9 as Unif(1,1); however, the main text (section 3.2.2) used Unif(10,10). Tables 2 and 4 have been updated to match the Unif(10,10) version.

**Table 2:** Overview of conducted MR analyses.

Group	Exposure ID	IVs selection	IVs adjustment	Bayesian MR
1	ukb-b-6591	*P < *5 × 10^−8^, *R*^2^ *< *0.001, 10,000 kb	No	No
2	ebi-a-GCST90000047	*P < *5 × 10^−8^, *R*^2^ *< *0.001, 10,000 kb	No	No
3	ebi-a-GCST90000046	*P < *5 × 10^−8^, *R*^2^ *< *0.001, 10,000 kb	No	No
4	ebi-a-GCST90000046	*P < *5 × 10^−8^, *R*^2^ *< *0.001, 10,000 kb	Yes	No
5	ebi-a-GCST90000046	*P < *5 × 10^−7^, *R*^2^ *< *0.01, 5000 kb	Yes	No
6	ebi-a-GCST90000046	*P < *5 × 10^−8^, *R*^2^ *< *0.001, 10,000 kb	No	Yes, prior ∼ N (0,1)
7	ebi-a-GCST90000046	*P < *5 × 10^−8^, *R*^2^ *< *0.001, 10,000 kb	No	Yes, based on Group 4 result
8	ebi-a-GCST90000046	*P < *5 × 10^−8^, *R*^2^ *< *0.001, 10,000 kb	No	Yes, based on Group 3 result
9	ebi-a-GCST90000046	*P < *5 × 10^−8^, *R*^2^ *< *0.001, 10,000 kb	No	Yes, prior ∼ Unif(−10,10)
10	ebi-a-GCST90000046	*P < *5 × 10^−8^, *R*^2^ *< *0.001, 10,000 kb	No	Yes, prior ∼ N (0.5,0.5^2^)

IV = instrumental variable, MR = Mendelian randomization.

**Table 4:** Bayesian analysis results for causal effect of AFS on ED.

Group	Prior Distribution	Posterior Estimates			Ȓ
		Mean (SD)	2.5% quantile	97.5% quantile	
6	*θ *∼ N (0, 1)	−0.290 (0.147)	−0.570	0.001	1.004
7	*θ *∼ N (−0.385, 0.208^2^)	−0.323 (0.118)	−0.555	−0.096	1.004
8	*θ *∼ N (−0.442, 0.172^2^)	−0.358 (0.111)	−0.575	−0.136	1.007
9	*θ *∼ Unif (−10,10)	−0.298 (0.147)	−0.578	−0.013	1.000
10	*θ *∼ N (0.5, 0.5^2^)	−0.261 (0.144)	−0.555	0.022	1.002

AFS = age at first sexual intercourse, ED = erectile dysfunction, Ȓ = Gelman-Rubin diagnostic statistic, SD = standard deviation.
